# Supplementary material for: Sustainable PHBH–Alumina Nanowire Nanocomposites: Properties and Life Cycle Assessment
Source: Polymers (Basel). 2022 Nov 20;14(22):5033. doi: 10.3390/polym14225033 (PMC9697647; doi:10.3390/polym14225033)
Supplement: Supplementary file 1 [file polymers-14-05033-s001.zip › polymers-2034341-supplementary.pdf]

polymers

Supplementary Information

Sustainable PHBH–Alumina Nanowire Nanocomposites: Properties and Life Cycle Assessment

Julen Ibarretxe [1], Laura Alonso [2], Nora Aranburu [3], Gonzalo Guerrica-Echevarría [3], Amaia Orbea [4] and Maider Iturrondobeitia [1,2,*]

1 eMERG Research Group, School of Engineering of Bilbao, Building II-I, University of the Basque Country (UPV/EHU), Rafael Moreno Pitxitxi 3, 48013 Bilbao, Spain
2 Life Cycle Thinking Group, Department of Graphic Design and Engineering Projects, University of the Basque Country (UPV/EHU), Plaza Ingeniero Torres Quevedo 1, 48013 Bilbao, Spain
3 POLYMAT and Department of Polymer and Advanced Materials: Physics, Chemistry and Technology, Faculty of Chemistry, University of the Basque Country UPV/EHU, Paseo Manuel de Lardizabal 3, 20018 Donostia-San Sebastián, Spain
4 CBET Research Group, Dept. of Zoology and Animal Cell Biology, Research Centre for Experimental Marine Biology and Biotechnology PiE and Science and Technology Faculty, University of the Basque Country (UPV/EHU). Sarriena z/g, 48940 Leioa, Spain
* Correspondence: maider.iturrondobeitia@ehu.eus; Tel.: +34-946014311

S1. TGA curves

Figures S1 and S2 show the TGA curves of the samples prepared by melt blending and solvent casting, respectively.

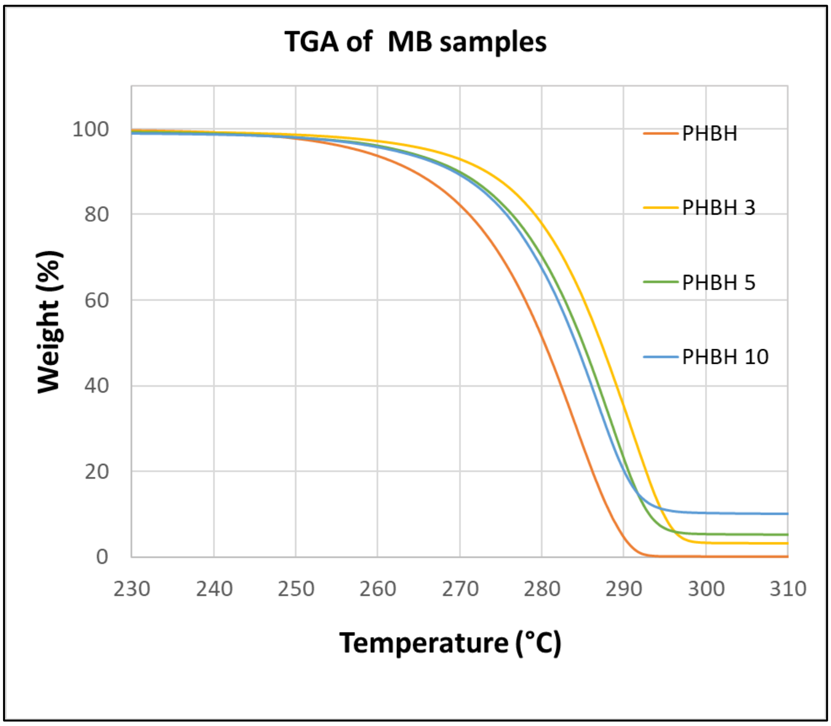

Figure S1. TGA curves of the samples prepared by melt blending.

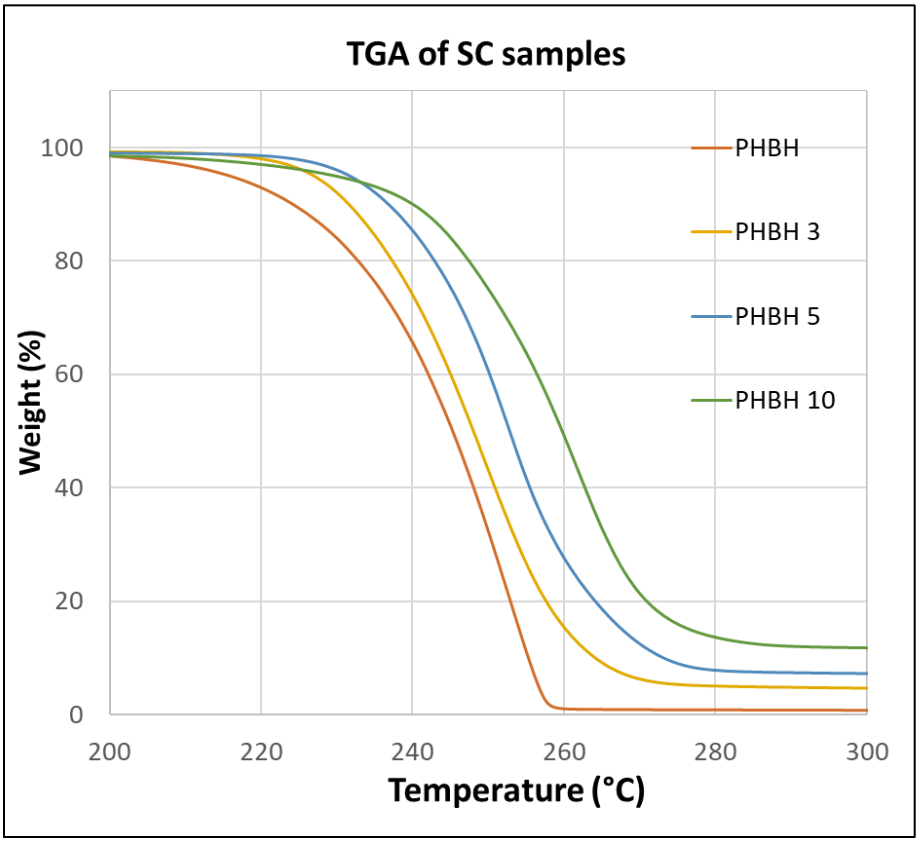

Figure S2. TGA curves of the samples prepared by solvent casting.

S2. Tensile test curves

Figure S3. shows the tensile test curves of the samples prepared by solvent casting.

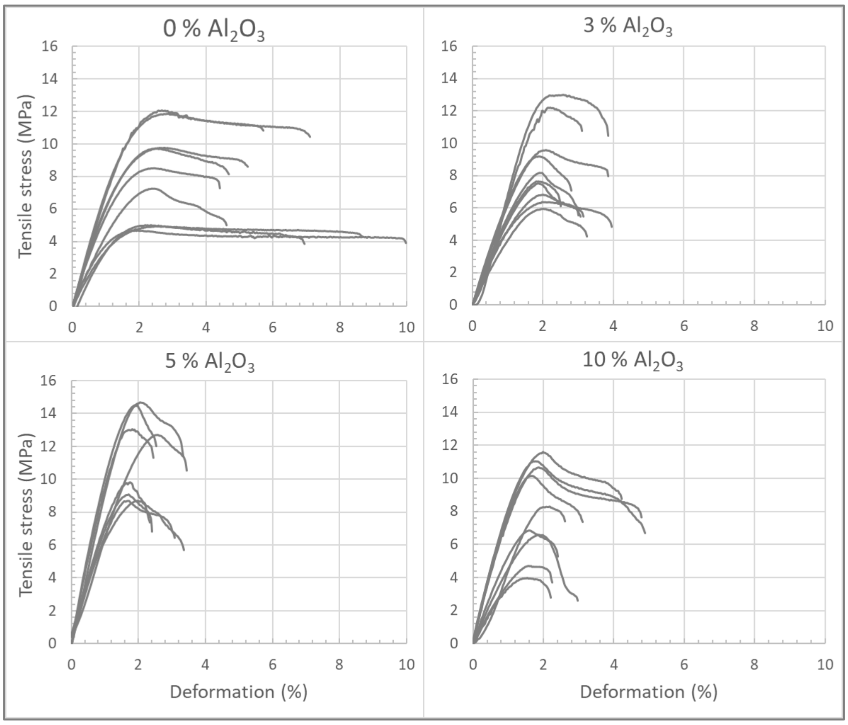

Figure S3. Tensile test curves of the samples prepared by solvent casting.

S3. Inventory tables

The functional unit is *the use of 1 Kg of polymer from production to processing, use and end of Life (EoL).* Cradle to Grave boundaries are used.

The studied cases are the following:

a- PHBH polymer: production, processing by melt blending and EoL composting.
b- PHBH polymer: production, processing by melt blending and EoL biodegradation in sea water.
c- Petrol based Polyamide (PA) production, processing by melt blending and processing and Landfill EoL.
d- PHBH polymer: production, processing by melt blending, EoL biodegradation in sea water.
e- PHBH polymer: production , processing by solvent casting, EoL biodegradation in sea water.

All the inventories are summarized in the following TablesS3.1-4.

Table S1. Modelling for the life cycle materials and energy flows for the production of 1 kg of polyhydroxybutyrate "PHB", (own source).

**Item**	**Amount**	**Unit**	**Source/provider**
	Input		
E	2.29	kWh	Own source
enzymes	0.47	kg	Enzymes production ǀ enzymes ǀ Cut-off, U - RER
Glycerol_98	17.0	kg	Elementary ǀ glycerol
Hydrochloric acid, without water, in 30% solution state	0.46	kg	Allyl chloride production, reaction of propylene and chlorine ǀ hydrochloric acid, without water, in 30% solution state ǀ Cut-off, U - RER
Hydrogen peroxide, without water, in 50% solution state	1.24	kg	Hydrogen peroxide production, product in 50% solution state ǀ hydrogen peroxide, without water, in 50% solution state ǀ Cut-off, U - RER
Sodium hypochlorite, without water, in 15% solution state	0.23	kg	Sodium hypochlorite production, product in 15% solution state ǀ sodium hypochlorite, without water, in 15% solution state ǀ Cut-off, U - RER
Tap water	28.8	kg	Market for tap water ǀ tap water ǀ Cut-off, U - Europe without Switzerland
	Output		
PHB	1.0	kg	
Methanol, from biomass	0.58	kg	Manufacture of coke and refined petroleum products/19a: Liquid and gaseous fuels from biomass

Sodium methoxide	0.56	kg	Manufacture of chemicals and chemical products/201:Manufacture of basic chemicals, fertilizers and nitrogen compounds, plastics and synthetic rubber in primary forms/2011:Manufacture of basic chemicals
Solid_waste	0.132	kg	Landfill of solid waste, at landfill site, landfill including landfill gas utilisation and leachate treatment
Wastewater, average	13.0	m3	Water supply; sewerage, waste management and remediation activities/37:Sewerage/370:Sewerage/3700:Sewerage
Water vapour	25.0	kg	Elementary flows/Emission to air/high population density

The energy required (named E) for all the processes has been calculated according to the existing processes available in ecoinvent 3.8 for the production of the petrochemical based conventional polymers (PP, PA, PET and PE) and the landfill processes which is an average of:

Energy, primary, from geothermal	Elementary flows/Resource/in ground
Energy, primary, from solar energy	Elementary flows/Resource/in air
Energy, primary, from water power	Elementary flows/Resource/in water
Energy, primary, from wind power	Elementary flows/Resource/in air

Table S2. Modelling for the life cycle materials and energy flows for the production –process and EoL- landfill of 1 kg of petroleum based PA.

**Item**	**Amount**	**Unit**	**Source/provider**
	Input		
POLYMER	2	kg	Nylon 66 granulate (PA 66), production mix, at plant
E	33.8	kWh	Own sorce
Transport, freight, lorry 16-32 metric ton, EURO5	38.18	t*km	Market for transport, freight, lorry 16-32 metric ton, EURO5 ǀ transport, freight, lorry 16-32 metric ton, EURO5 ǀ Cut-off, U - RoW
	Output		

Flow	Amount	Unit	Provider
Waste plastic	0.5	kg	Landfill of plastic waste, at landfill site, landfill including landfill gas utilisation and leachate treatment
Polypropylene fibres (PP)	0.5	kg	Material Landfill of plastic waste, at landfill site, landfill including landfill gas utilisation and leachate treatment and production/Plastics
Landfill	1	Kg	Landfill of plastic waste, at landfill site, landfill including landfill gas utilisation and leachate treatment + process-specific burdens, residual material landfill ǀ process-specific burdens, residual material landfill ǀ Cutoff, U - RoW

Table S3. Modelling for the life cycle materials and energy flows for the production –process and EoL- compost of 1 kg of PHB.

**Item**	**Amount**	**Unit**	**Source/provider**
		Input	
POLYMER	2	kg	PHB from table S6 (own source)
E	33.8	kWh	Own source
Transport, freight, lorry 16-32 metric ton, EURO5	38.18	t*km	Market for transport, freight, lorry 16-32 metric ton, EURO5 ǀ transport, freight, lorry 16-32 metric ton, EURO5 ǀ Cut-off, U - RoW
Land-compost	120	kg	Elementary flows/Land use/Land occupation
Oxygen	1	Kg	Elementary flows/Oxygen
		Output	
Flow	Amount	Unit	Provider
Waste plastic	0.5	kg	Landfill of plastic waste, at landfill site, landfill including landfill gas utilisation and leachate treatment
Carbon dioxide, biogenic	1.7	g	Elementary flows/Emission to air/low population density, long-term
Compost at plant	0.8	g	Compost at plant / GLO

Table S4. Modelling for the life cycle materials and energy flows for the production –process and EoL- biodegradation in sea water of 1 kg of PHB.

Item	Amount	Unit	Source/provider
	Input		
POLYMER	2	kg	PHB from table S6 (own source)
E	33.8	kWh	Own sorce
Transport, freight, lorry 16-32 metric ton, EURO5	38.18	t*km	Market for transport, freight, lorry 16-32 metric ton, EURO5 ǀ transport, freight, lorry 16-32 metric ton, EURO5 ǀ Cut-off, U - RoW
	Output		
Flow	Amount	Unit	Provider
Waste plastic	0.5	kg	Landfill of plastic waste, at landfill site, landfill including landfill gas utilisation and leachate treatment
Carbon dioxide, biogenic	1.2	g	Elementary flows/Emission to air/low population density, long-term
Water	0,3	g	Elementary flows/Emission

S4. Environmental impact assessment

Table S5. Results of the quantified environmental impacts for all the studied cases "a, b, c, d, e, and f" of 1 kg of PHB.

Indicator	PHB-sea degradation	PHB-compost	PA-landfill	PE-landfill	PP-landfill	PET-landfill
Fine particulate matter formation (kg PM2,5 eq)	2.95E-03	2.95E-03	3.06E-02	1.36E-02	1.60E-02	1.56E-02
Fossil resource scarcity (kg oil eq)	4.65E-01	4.65E-01	4.44E-02	4.44E-02	1.75E+00	4.44E-02
Freshwater ecotoxicity (kg 1,4-DCB)	7.06E-02	7.06E-02	3.66E-01	3.64E-01	4.17E-01	3.65E-01
Freshwater eutrophication (kg P eq)	6.00E-04	6.00E-04	4.23E-04	3.96E-04	8.20E-04	3.96E-04
Global warming (kg CO2 eq)	1.66E+00	1.66E+00	2.45E+01	9.81E+00	1.13E+01	1.09E+01
Human carcinogenic	7.36E+00	7.36E+00	6.11E+02	6.11E+02	6.17E+02	6.11E+02

toxicity (kg 1,4-DCB)						
Human non-carcinogenic toxicity (kg 1,4-DCB)	2.98E+02	2.98E+02	2.21E+03	2.16E+03	2.39E+03	2.16E+03
Ionizing radiation (kBq Co-60 eq)	1.72E-01	1.72E-01	4.94E+00	1.91E+00	1.96E+00	1.91E+00
Land use (m2a crop eq)	3.05E-03	1.30E-01	1.21E-02	1.21E-02	2.75E-02	1.21E-02
Marine ecotoxicity (kg 1,4-DCB)	3.79E+02	3.79E+02	3.27E+03	3.21E+03	3.50E+03	3.21E+03
Marine eutrophication (kg N eq)	5.50E-05	5.50E-05	5.65E-04	2.71E-04	3.04E-04	2.71E-04
Mineral resource scarcity (kg Cu eq)	5.50E-03	5.50E-03	6.93E-03	5.72E-03	1.18E-02	5.72E-03
Ozone formation, Human health (kg NOx eq)	3.64E-03	3.64E-03	3.29E-02	2.07E-02	2.51E-02	2.15E-02
Ozone formation, Terrestrial ecosystems (kg NOx eq)	3.68E-03	3.68E-03	3.37E-02	2.08E-02	2.56E-02	2.21E-02
Stratospheric ozone depletion (kg CFC11 eq)	1.32E+00	1.32E+00	4.78E-05	2.04E-06	2.49E-06	2.04E-06
Terrestrial acidification (kg SO2 eq)	5.70E-03	5.70E-03	1.02E-01	4.61E-02	5.24E-02	5.29E-02
Terrestrial ecotoxicity (kg 1,4-DCB)	4.37E+00	4.37E+00	1.04E+01	5.20E+00	6.95E+00	6.45E+00
Water consumption (m3)	-4.73E-01	-4.73E-01	3.05E-02	9.51E-03	3.42E-02	4.16E-02

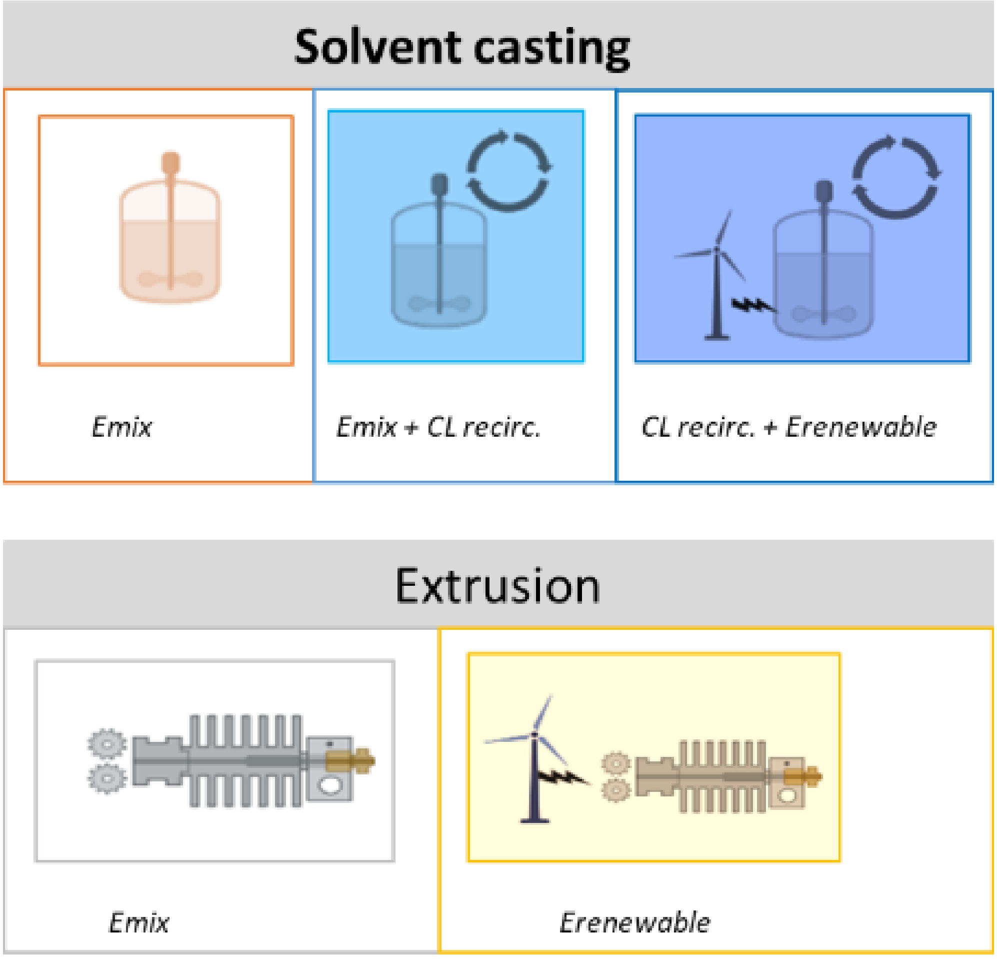

Figure S4. Visual summary of the sensitivity analysis to measure the effects of the type of energy sources and waste recirculation processes. *CL* stands for chloroform, which is the solvent used for the solvent casting processing technique.

Indicator	b-PHBH-sea water	a-PHBH-compost	c- PA-landfill	d- PHBH-alumina-extrusion	e- PHBH-alumina-solventAA	Unit
Fine particulate matter formation	0.01	0.01	2.29	0.01	0.10	kg PM2,5 eq
Fossil resource scarcity	1.38	1.38	0.00	1.39	31.74	kg oil eq
Freshwater ecotoxicity	0.14	0.14	0.15	0.14	2.79	kg 1,4-DCB
Freshwater eutrophication	0.00	0.00	0.02	0.00	0.02	kg P eq
Global warming	4.77	4.84	1512.84	4.84	47.76	kg CO2 eq
Human carcinogenic toxicity	15.36	15.36	1.65	15.51	259.28	kg 1,4-DCB

Human non-carcinogenic toxicity	612.13	612.30	2290.80	651.08	9684.81	kg 1,4-DCB
Ionizing radiation	0.45	0.48	325.68	0.48	7.72	kBq Co-60 eq
Land use	0.06	0.26	0.00	0.07	0.94	m2a crop eq
Marine ecotoxicity	777.64	777.87	3055.43	826.20	12391.30	kg 1,4-DCB
Marine eutrophication	0.00	0.00	0.03	0.00	0.00	kg N eq
Mineral resource scarcity	0.01	0.01	0.52	0.01	0.20	kg Cu eq
Ozone formation, Human health	0.01	0.01	3.40	0.01	1.11	kg NOx eq
Ozone formation, Terrestrial ecosystems	0.01	0.01	3.41	0.01	1.72	kg NOx eq
Stratospheric ozone depletion	0.00	0.00	0.00	0.00	0.00	kg CFC11 eq
Terrestrial acidification	0.01	0.01	7.82	0.01	0.19	kg SO2 eq
Terrestrial ecotoxicity	9.02	9.05	923.25	8.58	3827.86	kg 1,4-DCB
Water consumption	-0.95	-0.95	2.12	-0.83	0.34	m3

Table S6. Environmental impact results of the sensitivity analysis to measure the effects of energy sources and waste recirculation processes.

Indicator	Solvent Recovery CL	Solvent PHB	Extrusion PHB	Extrusion-renewable energy from wind	Solvent REcoveryCL + renewable energy
Fine particulate matter formation (kg PM2,5 eq)	0.025	0.099	0.007	0.005	0.023
Fossil resource scarcity (kg oil eq)	6.390	31.300	0.989	0.849	6.190
Freshwater ecotoxicity (kg 1,4-DCB)	0.548	2.790	0.143	0.132	0.534
Freshwater eutrophication (kg P eq)	0.005	0.018	0.001	0.001	0.004
Global warming (kg CO2 eq)	11.9	46.5	3.5	3.0	11.2
Human carcinogenic toxicity (kg 1,4-DCB)	61.0	259.0	14.9	13.3	58.1

Human non-carcinogenic toxicity (kg 1,4-DCB)	2260.0	9670.0	637.0	573.0	2170.0
Ionizing radiation (kBq Co-60 eq)	2.920	7.620	0.377	0.315	2.330
Land use (m2a crop eq)	0.238	0.940	0.066	0.058	0.223
Marine ecotoxicity (kg 1,4-DCB)	2890.0	12400.0	808.0	726.0	2780.0
Marine eutrophication (kg N eq)	0.000360	0.001260	0.000136	0.000120	0.000332
Mineral resource scarcity (kg Cu eq)	0.043	0.196	0.010	0.010	0.042
Ozone formation, Human health (kg NOx eq)	0.033	1.110	0.008	0.007	0.031
Ozone formation, Terrestrial ecosystems (kg NOx eq)	0.035	1.720	0.008	0.007	0.032
Stratospheric ozone depletion (kg CFC11 eq)	0.000009	0.000036	0.000003	0.000002	0.000009
Terrestrial acidification (kg SO2 eq)	0.050	0.187	0.012	0.010	0.046
Terrestrial ecotoxicity (kg 1,4-DCB)	20.900	3830.000	8.320	8.010	20.400
Water consumption (m3)	-0.642	0.336	-0.831	-0.833	-0.646

**Indicator**	**Solvent Recovery CL**	**Solvent PHB**	**Extrusion PHB**	**Extrusion-renewable energy from wind**	**Solvent REcoveryCL + renewable energy**
Fine particulate matter formation (kg PM2,5 eq)	2.49E-02	9.91E-02	6.55E-03	5.36E-03	2.32E-02
Fossil resource scarcity (kg oil eq)	6.39E+00	3.13E+01	9.89E-01	8.49E-01	6.19E+00
Freshwater ecotoxicity (kg 1,4-DCB)	5.48E-01	2.79E+00	1.43E-01	1.32E-01	5.34E-01
Freshwater eutrophication (kg P eq)	4.55E-03	1.79E-02	1.32E-03	1.09E-03	4.26E-03

Global warming (kg CO2 eq)	1.19E+01	4.65E+01	3.53E+00	3.03E+00	1.12E+01
Human carcinogenic toxicity (kg 1,4-DCB)	6.10E+01	2.59E+02	1.49E+01	1.33E+01	5.81E+01
Human non-carcinogenic toxicity (kg 1,4-DCB)	2.26E+03	9.67E+03	6.37E+02	5.73E+02	2.17E+03
Ionizing radiation (kBq Co-60 eq)	2.92E+00	7.62E+00	3.77E-01	3.15E-01	2.33E+00
Land use (m2a crop eq)	2.38E-01	9.40E-01	6.64E-02	5.83E-02	2.23E-01
Marine ecotoxicity (kg 1,4-DCB)	2.89E+03	1.24E+04	8.08E+02	7.26E+02	2.78E+03
Marine eutrophication (kg N eq)	3.60E-04	1.26E-03	1.36E-04	1.20E-04	3.32E-04
Mineral resource scarcity (kg Cu eq)	4.31E-02	1.96E-01	1.02E-02	9.95E-03	4.22E-02
Ozone formation, Human health (kg NOx eq)	3.30E-02	1.11E+00	7.85E-03	6.62E-03	3.06E-02
Ozone formation, Terrestrial ecosystems (kg NOx eq)	3.46E-02	1.72E+00	7.95E-03	6.71E-03	3.22E-02
Stratospheric ozone depletion (kg CFC11 eq)	9.08E-06	3.58E-05	2.69E-06	2.41E-06	8.61E-06
Terrestrial acidification (kg SO2 eq)	4.99E-02	1.87E-01	1.22E-02	1.04E-02	4.55E-02
Terrestrial ecotoxicity (kg 1,4-DCB)	2.09E+01	3.83E+03	8.32E+00	8.01E+00	2.04E+01
Water consumption (m3)	-6.42E-01	3.36E-01	-8.31E-01	-8.33E-01	-6.46E-01

Table S7. Environmental impact results of the sensitivity analysis to measure the effects of using different reinforcing particles to enhance the properties of the biopolymer.

**Indicator**	**Solvent PHB**	**Solvent PHB-aluminum**	**Solvent PHB-cellulose**	**Solvent PHB-clay**	**Solvent PHB-graphene**	**Solvent PHB-cellulose-natural**
Fine particulate matter	0.0991	0.0991	0.0998	0.0991	0.0992	0.0991

formation (kg PM2.5 eq)						
Fossil resource scarcity (kg oil eq)	31.300	31.300	31.400	31.300	31.300	31.300
Freshwater ecotoxicity (kg 1.4-DCB)	2.790	2.790	2.810	2.790	2.790	2.790
Freshwater eutrophication (kg P eq)	0.0179	0.0179	0.0180	0.0179	0.0179	0.0179
Global warming (kg CO2 eq)	46.50	46.50	46.70	46.50	46.50	46.50
Human carcinogenic toxicity (kg 1.4-DCB)	259.00	259.00	263.00	259.00	259.00	259.00
Human non-carcinogenic toxicity (kg 1.4-DCB)	9670.00	9670.00	9760.00	9670.00	9670.00	9670.00
Ionizing radiation (kBq Co-60 eq)	7.62	7.62	7.65	7.62	7.62	7.62
Land use (m2a crop eq)	0.9400	0.9400	0.9440	0.9400	0.9400	0.9400
Marine ecotoxicity (kg 1.4-DCB)	12400.0	12400.0	12500.0	12400.0	12400.0	12400.0
Marine eutrophication (kg N eq)	0.0013	0.0013	0.0013	0.0013	0.0013	0.0013
Mineral resource scarcity (kg Cu eq)	0.1960	0.1960	0.2030	0.1970	0.1960	0.1960
Ozone formation. Human health (kg NOx eq)	1.110	1.110	1.110	1.110	1.110	1.110
Ozone formation. Terrestrial ecosystems (kg NOx eq)	1.7200	1.7200	1.7200	1.7200	1.7200	1.7200
Stratospheric ozone depletion (kg CFC11 eq)	0.0000358	0.0000358	0.0000359	0.0000358	0.0000358	0.0000358
Terrestrial acidification (kg SO2 eq)	0.1870	0.1870	0.1880	0.1870	0.1870	0.1870

Terrestrial ecotoxicity (kg 1.4-DCB)	3830.0	3830.0	3830.0	3830.0	3830.0	3830.0
Water consumption (m3)	0.3360	0.3360	0.3380	0.3360	0.3360	0.3360
